# Use of ferrets for electrophysiologic monitoring of ion transport

**DOI:** 10.1371/journal.pone.0186984

**Published:** 2017-10-27

**Authors:** Niroop Kaza, S. Vamsee Raju, Joan M. Cadillac, John A. Trombley, Lawrence Rasmussen, Liping Tang, Erik Dohm, Kevin S. Harrod, Steven M. Rowe

**Affiliations:** 1 Department of Medicine, University of Alabama at Birmingham, Birmingham, Alabama, United States of America; 2 Gregory Fleming James Cystic Fibrosis Research Center, University of Alabama at Birmingham, Birmingham, Alabama, United States of America; 3 Animal Resources Program, University of Alabama at Birmingham, Birmingham, Alabama, United States of America; 4 Department of Anesthesiology, University of Alabama at Birmingham, Birmingham, Alabama, United States of America; Universidad de la Laguna, SPAIN

## Abstract

Limited success achieved in translating basic science discoveries into clinical applications for chronic airway diseases is attributed to differences in respiratory anatomy and physiology, poor approximation of pathologic processes, and lack of correlative clinical endpoints between humans and laboratory animal models. Here, we discuss advantages of using ferrets (*Mustela putorus furo)* as a model for improved understanding of human airway physiology and demonstrate assays for quantifying airway epithelial ion transport *in vivo* and *ex vivo*, and establish air-liquid interface cultures of ferret airway epithelial cells as a complementary *in vitro* model for mechanistic studies. We present data here that establishes the feasibility of measuring these human disease endpoints in ferrets. Briefly, potential difference across the nasal and the lower airway epithelium in ferrets could be consistently assessed, were highly reproducible, and responsive to experimental interventions. Additionally, ferret airway epithelial cells were amenable to primary cell culture methods for *in vitro* experiments as was the use of ferret tracheal explants as an *ex vivo* system for assessing ion transport. The feasibility of conducting multiple assessments of disease outcomes supports the adoption of ferrets as a highly relevant model for research in obstructive airway diseases.

## Introduction

Obstructive lung diseases such as cystic fibrosis (CF), chronic obstructive pulmonary disease (COPD), and asthma are characterized by a pronounced involvement of the airway epithelium [1]. These disorders collectively constitute a major cause of morbidity, mortality, and healthcare utilization [2], yet development of novel therapies to lessen disease burden is impeded by lack of animal models and techniques that are informative for elucidating disease biology, identifying new drug targets, and predicting the effect of drug candidates.

The search for better preclinical tools for obstructive lung disease has encouraged the development of higher-order mammals that have greater resemblance to humans than lower mammalian orders such as mice. Features important to model include the complex airway anatomy and prominent distribution of mucus-producing airway glands and goblet cells, which clearly impact epithelial function [3–5]. Indeed, while murine models of obstructive lung diseases have been useful for the identification of mechanisms related to inflammation, emphysema, fibrosis, and repair [6, 7], mice do not recapitulate some crucial features of airway diseases, like the development of spontaneous lung disease, which is a major limitation. Ion transport across the tracheal epithelium is an integral part of animal model characterization of airway diseases. One of the possible reasons murine models do not develop spontaneous chronic lung disease is the high expression of alternative cAMP-inducible chloride channels in the tracheal epithelium, unlike in higher order mammals like the ferret where CFTR is the primary cAMP-inducible chloride channel [8–10].

To improve translational utility, ferrets are proving advantageous. Recently, a CF ferret model was developed that exhibits features highly reminiscent of human lung disease and, if GI obstruction can be resolved by genetic or pharmacologic manipulation, could be highly informative due its size and utility for interventional studies [10, 11]. Ferrets are also commonly used to model viral infections of the respiratory system, which clearly impact epithelial function [12–14], and are amenable to complex research maneuvers to induce lung disease (i.e. cigarette smoke or other environmental exposures). For instance, ferrets have recently been demonstrated to generate chronic bronchitis, in addition to emphysema, upon cigarette smoke exposure, providing notable opportunities to examine the biology of COPD [15].

Despite the emerging strengths of ferrets in modeling airway disease, techniques to interrogate the epithelial biology of ferret airways have not yet been fully developed. As such, we have developed novel assays of airway electrophysiology in ferrets to investigate disease mechanisms relevant to obstructive airway disease research. In this manuscript, we demonstrate reliable conduct of transepithelial potential difference (PD) across the nasal and lower airway ferret epithelium as well as within-subject reproducibility of these measures, thus supporting further investigations using ferrets, including interventional studies. In addition, we describe and implement tissue culture methods for conducting air-liquid interface cultures (ALI) of ferret bronchial epithelial cells, which have potential utility for *in vitro* validation of physiologic mechanisms and for identifying pharmacologic and molecular agents, as have been successfully implemented in other species such as swine [16–18].

## Methods

### Animal welfare and approvals

This study was carried out in strict accordance with the recommendations in the Guide for the Care and Use of Laboratory Animals of the National Institutes of Health. The protocol was approved by the Institutional Animal Care and Use Committee (IACUC) of the University of Alabama at Birmingham (APN: 09681). All *in vivo* procedures were performed under anesthesia, and all efforts were made to minimize suffering. Ferrets were euthanized by exsanguination from cardiac puncture following an injection of ketamine and dexmedetomide at the end of the study to assure appropriate analgesia and anxiolysis.

### Transepithelial nasal potential difference (NPD) measurement

**Fig 1:**
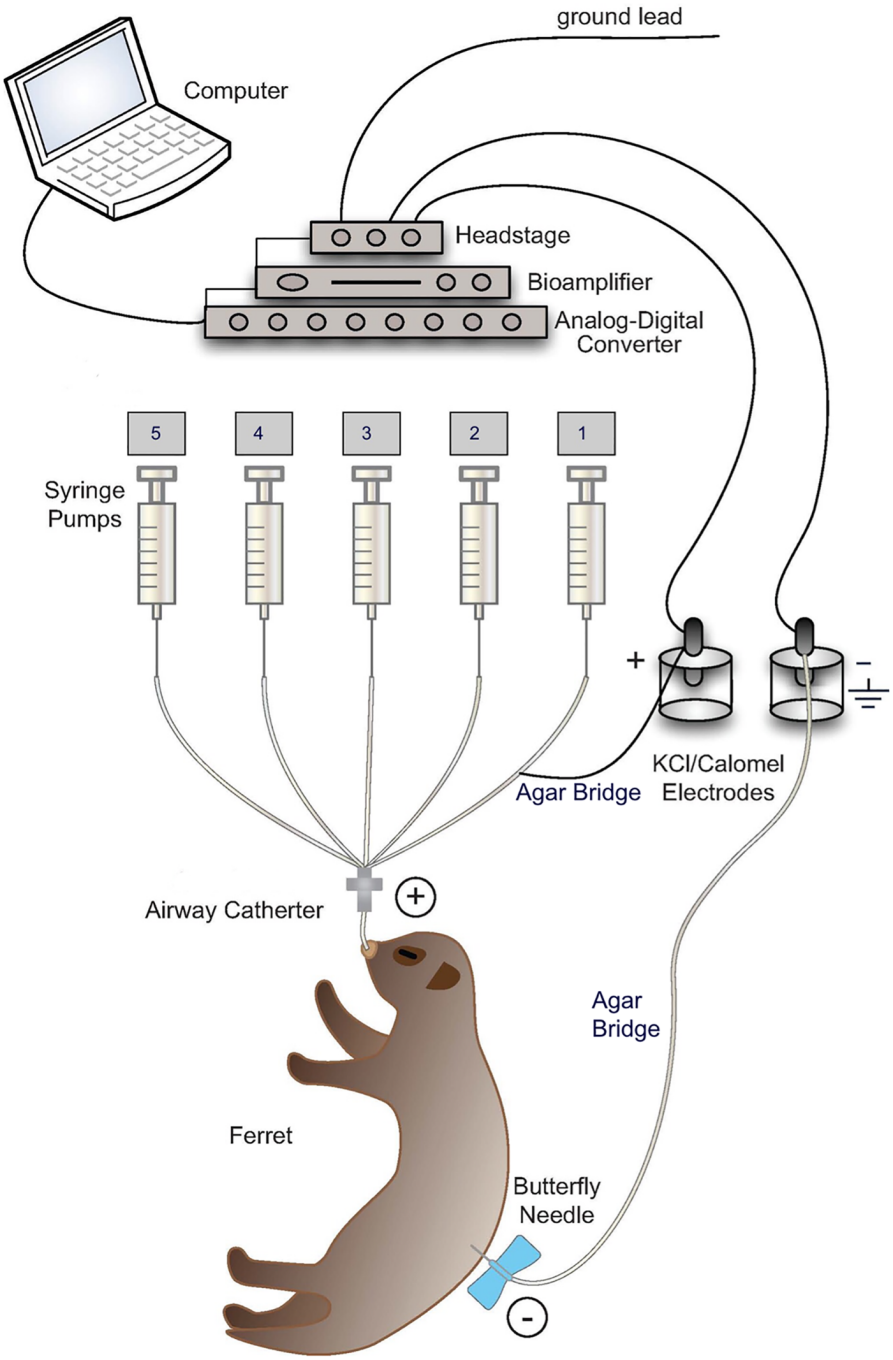
Schematic of laboratory set up for potential difference (PD) measurements in ferrets. Cartoon representing hardware and electrical connections representing PD apparatus modified from the human instrument recommended by the standard operating procedure of the CF Therapeutics Development Network and the European Clinical Trial Network [19]. Ground Lead: Connects electrical apparatus to ground for reference from which voltage is measured. Headstage: Connects electrode inputs to bioamplifier and safely isolates from wall AC current. Bioamplifier: Serves as voltmeter to measure PD. Analog-Digital Converter: Converts analog signals to digital form. K/Cl Calomel Electrodes: Measure PD. Agar Bridge: Connects positively charged (+) calomel electrodes to ferret nasal epithelium or lower airway epithelium through Airway Catheter and negatively charged (-) calomel electrodes to subcutaneous tissue through Butterfly Needle. Syringe Pumps: Deliver test solutions perfused sequentially from pump 1 to 5 with a flow rate of 4ml/hr (see S1 Table). The PD changes are observed and recorded on the computer using LabChart 7.0 software. Following the procedure, the nasal cannula and subcutaneous needle were removed, and anesthesia reversed with atipamezole (5 mg/kg body weight, IM) delivered at an equal volume as dexmedetomidine. All ferrets recovered within 10–15 min, and remained on a warming pad until aroused and moving. Supplemental oxygen was not required during the NPD procedures in our experience, however supplemental oxygen was delivered during recovery from anesthesia.

Transepithelial NPD in ferrets was measured using an apparatus previously used in human studies (Fig 1) and following the standard operating procedure of the CF Therapeutics Development Network and the European Clinical Trial Network [19]. Hardware included a 4/30 PowerLab Analog-Digital Converter (AD Instruments, Colorado Springs, CO), BMA-200 AC/DC Bioamplifier (CWE, Ardmore, PA), ISO-Z isolation headstage for BMA-200 (CWE, Ardmore, PA), and a PC operating LabChart 7.0 software. PD was measured with Accumet miniature KCl calomel reference electrodes (Fisher Scientific, Cat. No:13-620-79) coupled with agar bridges to the nasal and subcutaneous compartments and to perfusion pumps to perfuse reagents into the nasal cavity (five in total, one for each drug infusion; WPI, Sarasota, FL). Other equipment included PE50 tubing and connectors (Braintree Scientific, Boston, Ma), an illuminating rhinoscope, heating pads, and disposables.

Adult male and female ferrets weighing between 0.6–1.8 kg were anesthetized with a drug combination of dexmedetomidine (0.08–0.2 mg/kg, IM) and ketamine (2.5–5 mg/kg, IM), and then placed on a heated operating table until a deep state of anesthesia was reached. Once sedation was achieved, ophthalmic petroleum jelly was applied to the eyes to minimize drying. For NPD measurements, topical 2% lidocaine gel was applied to the outlet of the left nare to inhibit sneeze response, which is particularly prominent in ferrets even under deep sedation. With the injectable anesthetic combination, ferret respirations are not substantially depressed and do not require assisted ventilation, although supplemental oxygen via blow-by can be provided, if required.

Five minutes following lidocaine application, ferrets were sufficiently somnolent and the nasal outlet anesthetized to permit cannulation with a PE-90 cannula pulled to a tip diameter of 0.2 mm and inserted 6–9 mm into the left nostril, just lateral to the nasal septum (S1 Fig). The nasal cannula was connected to cathode electrode in 3 M KCl via an agarose bridge. An IV needle (30G) filled with 1% agarose in 150 mM NaCl solution was placed under the skin and connected to anode electrode in 3 M KCl solution to close the measuring circuit (Fig 1). Following acquisition of a viable baseline potential with Ringer’s USP, test solutions were perfused serially to measure the change in PD attributable to sodium transport through epithelial sodium channel (ENaC) and chloride transport through cystic fibrosis transmembrane conductance regulator (CFTR). Details of each solution and its composition are described in S1 Table. The perfusion pumps were set up in the same sequence as listed in S1 Table and were operated in numerical order. The solutions were dispensed from a 1 mL Becton Dickson syringe and each drug was perfused at a steady flow rate of 4 ml/hr for at least five minutes or until a stable signal was achieved.

### Lower airway potential difference (LAPD) measurement

Ferrets were sedated as described above. Following the application of the eye ointment, ferrets were intubated using a laryngoscope and a 4.3mm O.D Mallinckrodt oral/nasal uncuffed endotracheal tube (Covidien, Mansfield, MA). After confirming the position of the endotracheal tube by using a Nellcor pediatric colorimetric CO_2_ detector (Covidien, MA), a PE-60 cannula was inserted into the tube lumen until it passed through the tube end and contacted the epithelial lining of the lower airway. Like for NPD, the measuring circuit was assessed with a subcutaneous anode. Changes in PD were measured using the NPD protocol. Like the NPD protocol, LAPD procedures did not pose serious safety threats even with repeated conduct. All animals that underwent the procedure survived, with 100% recovery from the anesthetic regimen.

### Primary culture of ferret airway epithelial cells

Ferret airway epithelial cells were grown as primary cultures following techniques used in primary culture of human airway epithelial cells [20, 21]. Airway epithelial cells were dissociated from the trachea, grown at ALI, and used in short-circuit current (Isc) experiments after terminal differentiation. Briefly, trachea was excised from a euthanized ferret and debrided immediately following excision. Trachea were then washed twice in Minimum Essential Media (MEM) containing 0.5 mg/ml DTT (Sigma-Aldrich, St. Louis, MO) and 25 U/ml DNAse I (Roche, Basel, Switzerland). Following the washes, trachea was placed in dissociation media containing MEM, 2.5 U/ml DNAse I, 100 μg/ml ceftazidime, 80 μg/ml tobramycin, 1.25 μg/ml amphotericin B, and 4.4 U/ml pronase (Sigma-Aldrich) for 24–36 h at 4°C. Loosened airway epithelial cells were then expanded in growth media containing BEGM (LONZA, Basel, Switzerland) supplemented with an additional 10 nM all trans-retinoic acid (Sigma-Aldrich) every 24 h. Once cells were 80–90% confluent, they were seeded on Snapwell 1.13 cm^2^ permeable supports (1×10^6^cells/filter; Bayer, Pittsburgh, PN) or Costar 0.4 μm permeable supports (5×10^5^ cells/filter; Bethesda, MD) after coating with NIH 3T3 fibroblast-conditioned media, and grown in differentiating media containing DMEM/F12 (Invitrogen, Carlsbad, California), 2% Ultroser-G (Pall, New York, NY), 2% Fetal Clone II (Hyclone, Logan, UT), 2.5 μg/ml Insulin (Sigma-Aldrich), 0.25% bovine brain extract (LONZA), 20 nM hydrocortisone (Sigma-Aldrich), 500 nM Triodothyronine (Sigma-Aldrich), 2.5 μg/ml transferrin (Invitrogen), 250 nM ethanolamine (Sigma-Aldrich), 1.5 μM epinephrine (Sigma-Aldrich), 250 nM phosphoetheanolamine (Sigma-Aldrich), and 10 nM all trans-retinoic acid until terminally differentiated. Only first and second passage cell cultures with transepithelial electrical resistance (TEER) of more than 100 ohms/cm^2^ were considered for electrophysiological studies. Cells were fixed with standard histologic techniques for microscopy [12].

### Short-circuit current measurements on cultured primary ferret bronchial epithelial cells and *ex vivo* ferret trachea

Short-circuit current (Isc) was measured under voltage clamp conditions using MC8 clamps and P2300 Ussing chambers (Physiologic Instruments, San Diego, CA). ALI monolayers were initially bathed on both sides with identical Ringer’s solutions containing (in mM) 115 NaCl, 25 NaHCO_3_, 2.4 KH_2_PO_4_, 1.24 K_2_HPO_4_, 1.2 CaCl_2_, 1.2 MgCl_2_, and 10 D-glucose (pH 7.4). Bath solutions were vigorously stirred and gassed with 95%O_2_∶5% CO_2_. Isc was obtained using an epithelial voltage clamp (Physiologic Instruments). A one-second three-mV pulse was imposed every 10 seconds to monitor resistance calculated using Ohm’s law. Where indicated, the mucosal bathing solution was changed to a low Cl^−^ solution containing 1.2 NaCl and 115 Na^+^ gluconate, and all other components as above. This was followed by the CFTR agonist forskolin as indicated with minimum five-min observation at each concentration. GlyH101 (20 μM final concentration) was added to the mucosal bathing solution at the end of experiments to block CFTR-dependent Isc. All chambers were maintained at 37°C.

Tracheal Isc studies to assess ion transport across the epithelium were conducted on freshly harvested trachea from euthanized ferrets. Isc measurements in ferret airway explants were conducted following dissection of the mucosal layer and were performed as in ALI monolayers, except for use of P2307 tissue mounts. Amiloride (100 μM) was added to block residual Na^+^ current and was followed by a low Cl^−^ solution containing 1.2 NaCl and 115 Na^+^ gluconate. The CFTR agonist forskolin and antagonist GlyH101 were added to the mucosal bathing solution, where indicated, to respectively activate and thereafter block CFTR-dependent Isc.

### Statistics

Descriptive statistics (mean, SD, and SEM) were compared using 2-sided Student’s *t*test or 2-way ANOVA, as appropriate. Post-hoc tests for multiple comparisons following ANOVA were calculated using Fisher’s least significant difference only if ANOVA was significant. All statistical tests were 2-sided and were performed at a 5% significance level (i.e., α = 0.05) using GraphPad Prism software. Error bars designate SEM unless indicated otherwise.

## Results

### Transepithelial NPD tracings in ferrets resemble human tracings

NPD is the voltage across the nasal epithelium, representing the sum of the membrane potentials of the outer epithelial membrane. NPD can be used to evaluate ion transport *in vivo*, including the contributions of CFTR and ENaC, and thus is a particularly valuable tool for diagnosis and assessment of CF and other airway diseases characterized by chloride and sodium ion transport defects [22]. Notably, previous studies in animals and humans indicate that PD measurements across the nasal epithelium are generally reflective of the PD of the lower airway epithelium [23, 24].

**Fig 2:**
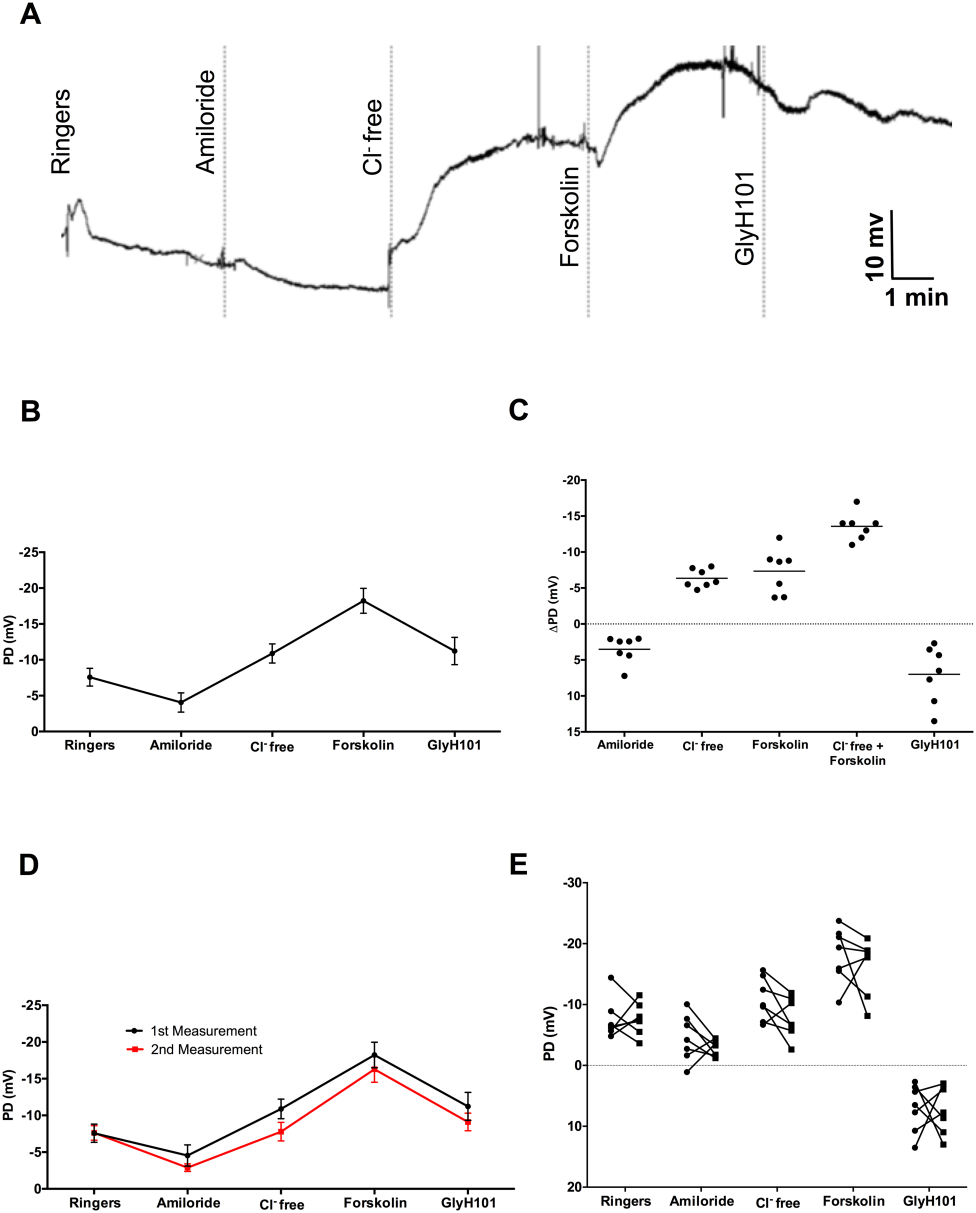
Measurement of nasal potential difference (NPD) in wild type ferrets. **A**: Representative NPD tracing in ferrets using the apparatus described in Fig 1. Under deep sedation, PD changes following perfusion with Ringer’s solution, Ringer’s solution with amiloride (100 μM), chloride-free solution, chloride-free with forskolin (20 μM), and chloride-free with CFTR-specific inhibitor GlyH101 (10 μM). **B**: Summary tracing of mean PD measurements (mean ± SEM) in ferrets following infusion of these 5 sequential reagents, N = 7. Ion channel activity of CFTR is quantified by PD changes in response to chloride-free + forskolin or by GlyH101 inhibitor. Similarly, ENaC activity is attributed to PD changes following amiloride treatment. **C**: Change in NPD (ΔPD) measurements from the same cohort of male and female adult, wild type ferrets plotted as individual measurements. **D**: Summary tracing of PD measurements (mean ±SEM) in male and female, adult, wild type ferrets acquired at two different times to validate reproducibility, N = 7. No discernable differences in ENaC or CFTR activity were found over 7 weeks. **E**: PD measurements of each animal subject plotted individually for each perfusate at two time points conducted 7 weeks apart.

To establish techniques to measure airway electrophysiology in ferrets, we developed and tested a modified method to assess ferret NPD. Fig 2A depicts a representative ferret NPD tracing, which demonstrates all the classical features of tracings observed in humans, including 1) a stable baseline PD that is inhibited by amiloride perfusion; 2) the subsequent hyperpolarization of the membrane upon sequential perfusion with chloride-free Ringers solution and chloride-free Ringers plus forskolin; and 3) partial (i.e. incomplete) blockade of CFTR-dependent hyperpolarization by the CFTR inhibitor GlyH101. These features held when PD measurements were averaged across all ferrets tested (Fig 2B). The average change in epithelial PD (ΔPD) (mean ± SD) with the addition of chloride-free Ringers plus forskolin (Δchloride-free forskolin),representative of the change in CFTR-dependent chloride transport,in ferrets was -13.7 ± 2 mV, while in healthy humans it is in the -29 ±10 mV range (22); other ΔPD measurements from the same ferrets are shown in Fig 2C and Table 1. ΔGlyH101 was 7.0 ± 3.9 mV, and indicated specificity of ΔPD as a metric of CFTR function. The change in PD upon amiloride perfusion, which signifies the change in ENaC-mediated epithelial sodium transport, was 3.5 ± 1.9 mV, and is comparably diminished in magnitude as compared to human values 13 ± 4 mV [22].

**Table 1:** Potential difference measurements across nasal epithelium.

Treatment	Mean PD (mV)	SD
Ringer’s	-7.57	3.26
ΔAmiloride	3.52	1.88
ΔChloride-free	-6.35	1.28
ΔForskolin	-7.34	3.10
ΔChloride-free + ΔForskolin	-13.69	2.03
ΔGlyH101	7.00	3.97

Importantly, ion transport measurements were repeatable (Fig 2D and 2E, and Table 2); the within subject repeatability of Δchloride-free froskolin was 0.3 ± 5.3 [mean ± SD], with no serious safety concerns. The anesthetic regimen achieved 100% recovery following multiple sessions of sedation, and multiple ferrets have been successfully evaluated during repeated sessions conducted as few as three days apart, permitting longitudinal analyses over the course of chronic studies. Together, these data indicate that NPD tracings in ferrets are similar to those seen in humans, and can be reliably and safely measured in a repeatable fashion.

**Table 2:** Differences in potential difference at separate measurements.

Treatment	Mean of PD differences between measurements	SD of differences
ΔAmiloride	1.21	2.42
ΔChloride-free	1.44	3.09
ΔForskolin	1.15	5.50
ΔChloride-free + ΔForskolin	0.28	5.29
ΔGlyH101	0.18	6.93

### Sensitivity of ferret NPD to pharmacological intervention

The ability to repeatedly assess NPD in the same animal enabled the calculation of sample sizes required for evaluating a significant difference in PD measurements in an experimental cohort of animals (Table 3). Sample sizes for detecting within-subject changes in PD are quite feasible for reasonable effect magnitudes.

**Table 3:** Sample size calculations to detect significant change in nasal potential difference if using the ferret model.

True Difference in PD (mV)	% Difference	Sample size to attain 80% power	Sample size to attain 90% power
2.7	20	31 pairs	41 pairs
4.1	30	15 pairs	19 pairs
5.5	40	9 pairs	12 pairs
6.8	50	7 pairs	8 pairs

**Fig 3:**
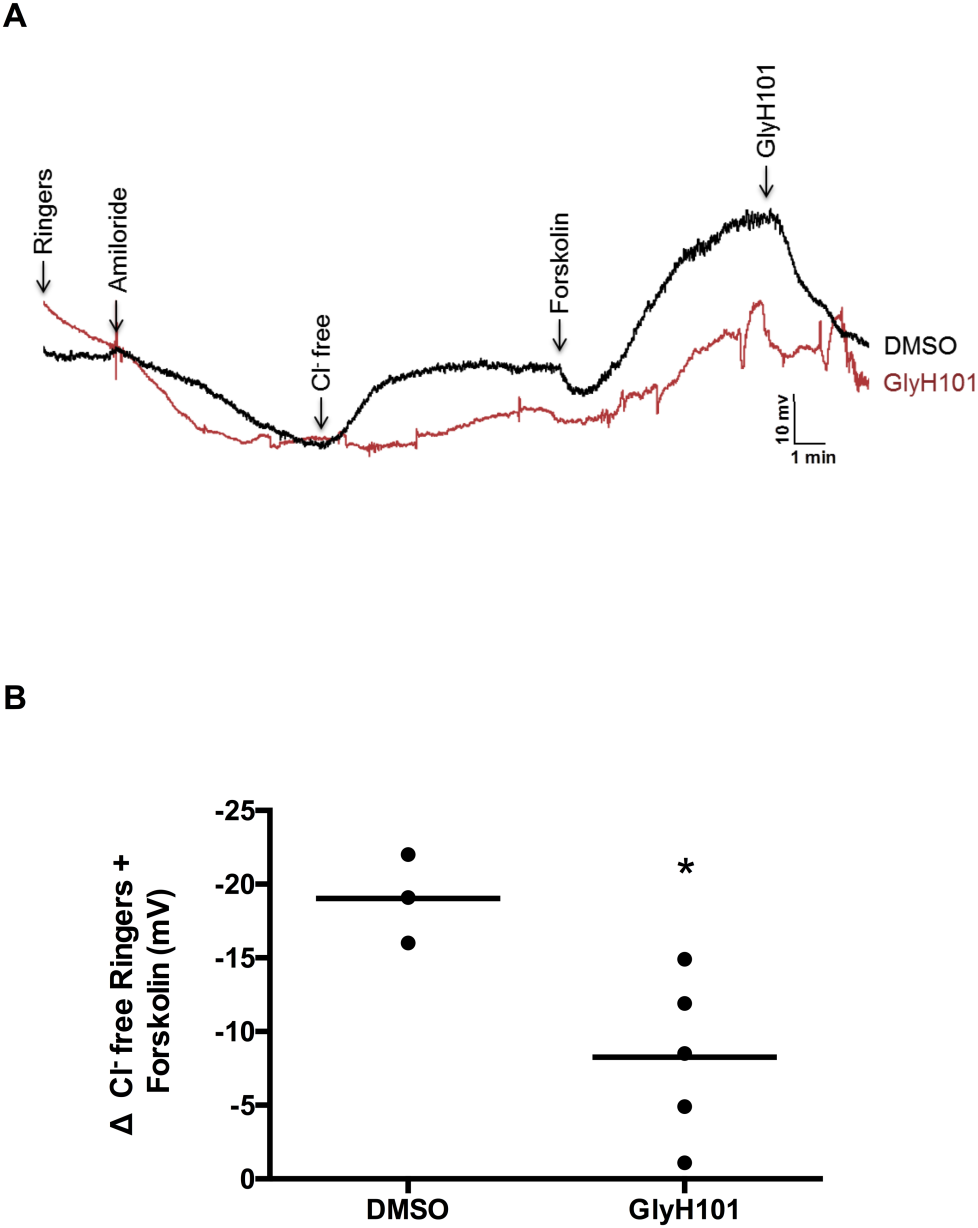
Reproducibility of nasal potential difference (NPD) assays in ferrets. **A**: Representative NPD tracing in ferrets one hour after acute intranasal administration with either vehicle (DMSO 0.001% in saline) or CFTR-specific inhibitor GlyH101 (10μM). **B**: Summary of CFTR-dependent PD differences in vehicle and GlyH101-treated ferrets. N = 4, * P<0.05.

As a proof of concept for demonstrating a within-ferret change in PD upon an intervention, we tested the sensitivity of NPD as a readout of diminished CFTR function, measuring PD 30 min following intranasal instillation with GlyH101 (10 μM) as compared to intranasal vehicle control (DMSO, 0.001%) in ferrets (N = 4). Results indicated that NPD was sensitive to inhibition; as the primary measure of CFTR activity, Δchloride-free forskolin, was significantly diminished (Fig 3A and 3B). All ferrets recovered following acute administration of GlyH101 and were adequately tolerant of repeat analyses.

### Lower airway potential difference (LAPD) measurements in ferrets

**Fig 4:**
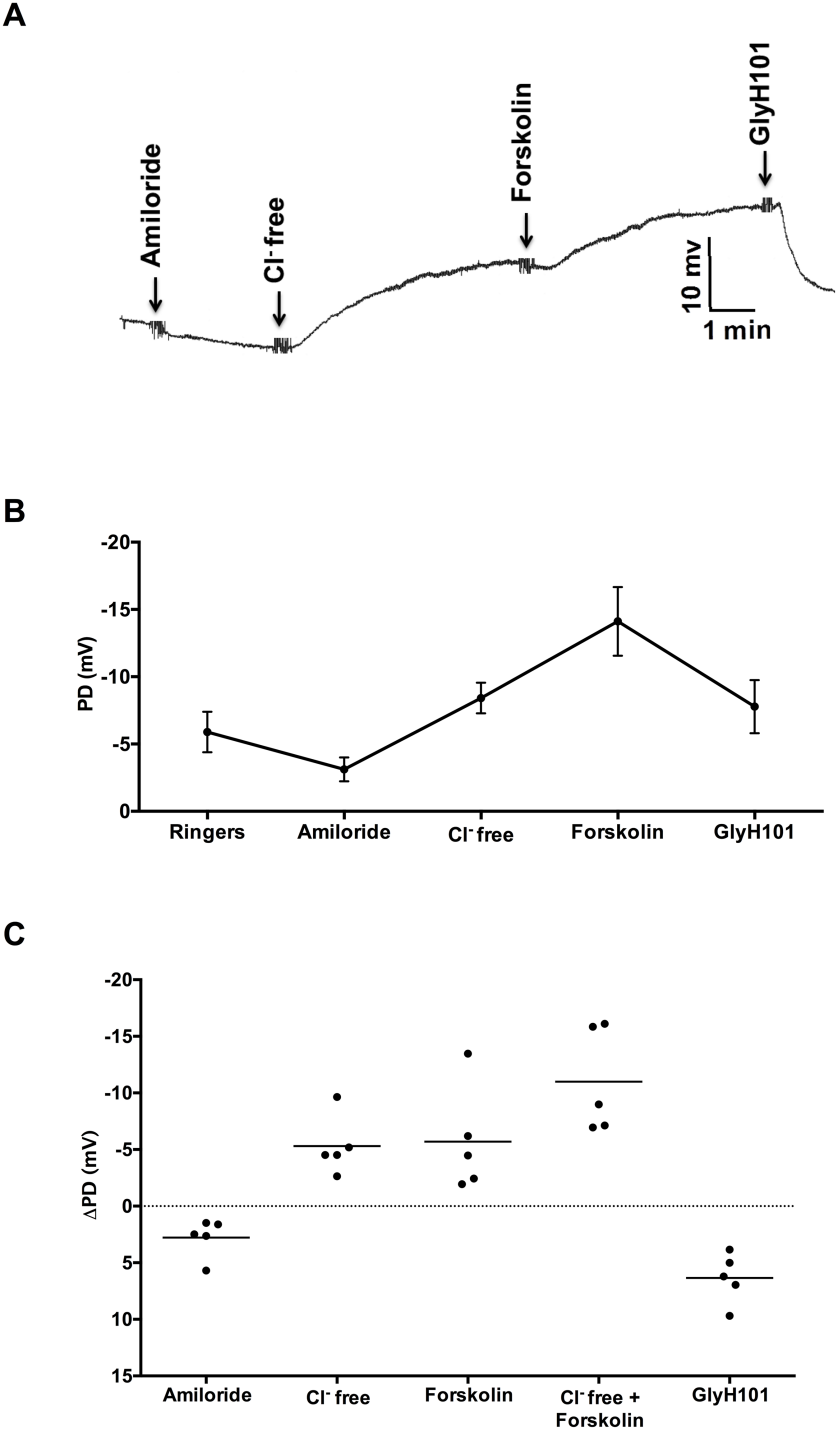
Measurement of lower airway potential difference (LAPD) in wild type ferrets. **A**. Representative LAPD tracing in a sedated intubated ferret indicating PD changes following perfusion with Ringer’s solution, amiloride (100 μM), chloride-free, chloride-free + forskolin (20 μM), and CFTR-specific inhibitor GlyH101 (10 μM); N = 5. **B**: Summary tracing of PD changes in ferrets following infusion of the 5 sequential reagents, N = 5. **C**: Change in LAPD measurements from 5 (male and female) adult, wild type ferrets.

PD measurements across the epithelial membrane in the lower airway provide a measure of the ion transport of the lung, and are potentially useful to evaluate inhaled interventions. Therefore, we developed a protocol to assess LAPD in ferrets, as this measure may be more informative than NPD for testing of therapies delivered specifically to the lungs [25, 26]. The representative ferret LAPD tracing (Fig 4A) and summary data (Fig 4B) demonstrate expected changes in PD upon sequential perfusion of test solutions. Average LAPD Δchloride-free froskolin was -11.0 ± 4.6 mV, and other PD measures (Fig 4C, Table 4) were also consistent with NPD, reflective of the similarities between the two outcome measures.

**Table 4:** Potential difference measurements across lower airway epithelium.

Treatment	Mean PD (mV)	SD
Ringer’s	-5.89	3.36
ΔAmiloride	2.77	1.70
ΔChloride-free	-5.30	2.60
ΔForskolin	-5.69	4.66
ΔChloride-free + ΔForskolin	-11.00	4.61
ΔGlyH101	6.33	2.21

### ALI cultures of ferret airway epithelial cells provide a comparable *in vitro* model system to *ex vivo* tracheal tissue for electrophysiologic studies

**Fig 5:**
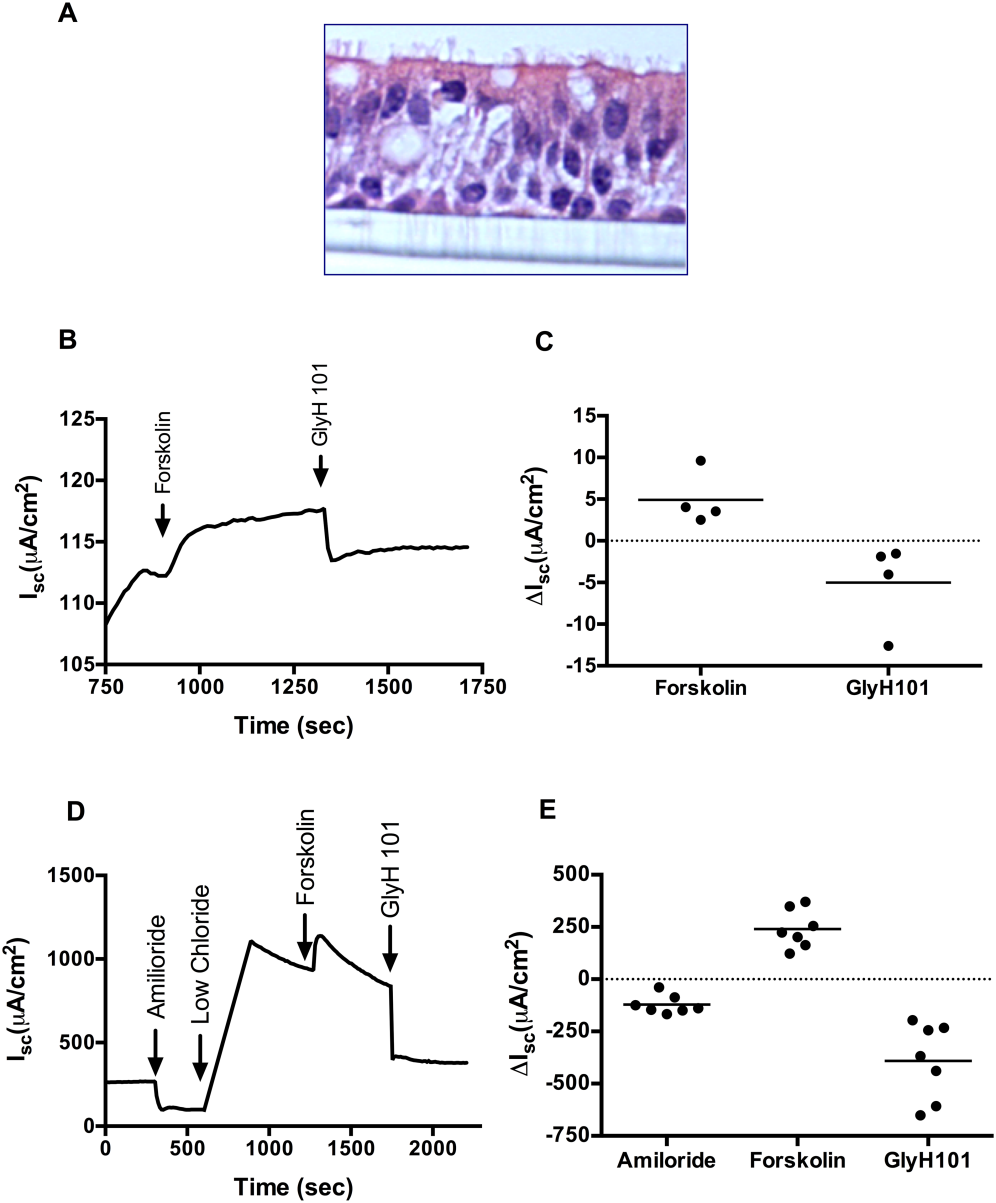
Measurement of short-circuit current (Isc) in cultured ferret bronchial epithelial cells and in explants of ferret trachea. **A**: Representative hematoxylin and eosin stained image of a well-differentiated ferret bronchial epithelial cells grown at air-liquid interface. **B**: Representative Isc tracings of primary ferret bronchial epithelial cells sequentially exposed to forskolin (10 μM), followed by GlyH101 (20 μM) in the setting of amiloride (100 μM) and a chloride secretory gradient. **C**: Summary data of Isc measurements from different ALI cultures indicating stimulated Isc following acute addition of forskolin or GlyH101. N  =  4/condition. **D**: Representative Isc tracing of an *ex vivo* ferret trachea sequentially exposed to Ringer’s, chloride-free, forskolin (20 μM), followed by GlyH101 (20 μM) in the setting of amiloride (100 μM). **E**: Summary data of Isc measurements from different trachea showing stimulated Isc following acute addition of amiloride, forskolin, or GlyH101. N = 7/condition.

Asymmetric distribution of proteins on polarized epithelium renders the apical properties of these cells distinctly different from their basolateral properties. Primary human airway epithelial cells grown on semi-permeable filters at ALI have been used extensively as a physiologically relevant *in vitro* model system [27]. To extend the advantages of ALI cultures to the ferret model, we isolated tracheobronchial epithelia from ferret trachea and investigated their eletrophysiological responses. We estimate over 1 x 10^8^ cells are available from a single ferret trachea prep, based on an area of 39 cm^2^ for a ferret trachea of 15 cm length & cell density comparable to that observed in ALI cultures. On histology, ALI cultures of ferret bronchial epithelial cells resembled primary human airway epithelial cells (Fig 5A). We tested CFTR activation with low chloride forskolin (10 μM), in the presence of amiloride (100 μM) to block ENaC channels, followed by CFTR inhibition with GlyH101 (20 μM). As shown in representative Isc tracing, increased anion transport occurred with addition of forskolin, which was immediately followed by CFTR inhibition with GlyH101, indicating CFTR specificity (Fig 5B). Summary data (Fig 5C) shows a mean Δforskolin Isc of 4.9 (± 1.6) μA/cm^2^ and a mean ΔGlyH101 Isc of -5.0 (± 2.6) μA/cm^2^.

To evaluate the findings in comparison to native tissue, we tested whether the same response was also observed in excised ferret airway explants. As seen in ferret ALI monolayers, ferret tracheal Isc tracings showed acute CFTR activation with both low chloride and forskolin as well as significant inhibition with GlyH101 (Fig 5D); summary dot showed stimulus with forskolin produced a mean ΔIsc of 240.4(± 34.6 μA/cm^2^ and GlyH101 induced a mean reduction of -391.4 ± 69.46 μA/cm^2^ (Fig 5E). Overall, these data demonstrate that ferret ALI cultures successfully recapitulate characteristics and functions comparable to *in vivo* and *ex vivo* systems.

## Discussion

Here we demonstrate multiple methods for conducting electrophysiologic studies of the airway epithelium of ferrets, a species of increasing importance in airway biology research. First, we establish a method for measuring nasal transepithelial PD—including the within-subject repeatability of the measurements—enabling estimation of ENaC, CFTR, and alternative chloride channel activity. A particular strength of the technique established here is that the methods are almost identical to those performed in humans, and include the advantages of a non-perfusion catheter, allowing for suitable translation [19, 28]. Sample size estimates suggest that a reasonable number of animals are required to detect meaningful changes in CFTR-dependent PD with an intervention or treatment, allowing for appropriately powered studies. Using these principles, we validate this concept showing blockade of CFTR activity upon nasal instillation of the CFTR inhibitor GlyH101. Overall findings suggest that it will be feasible to test for an electrophysiological effect in ferrets *in vivo*, such as the noxious effects of inhalational exposures, or the pharmacological benefit manifest by novel small molecules that activate ion channels [15]. They also render it possible to screen molecular reagents (e.g antibodies) and pharmacologic entities for their use in *in-vivo* studies with ferrets.

In a related effort, we also extend transepithelial PD measurements to the lower airway of ferrets, and demonstrate feasibility of the technique in sedated ferrets. Just as our laboratory and others have measured PD in the lungs of patients with CF or COPD [24, 25], we used a similar method to measure ferret LAPD. Although blinded placement of the catheter was required, the long trachea of ferrets provide reasonable assurance of position by measurement of the catheter, where we found consistent values between animals. As expected and observed in humans, the amiloride- and forskolin-sensitive PD in the lung was slightly lower than in the nose, probably reflective of channel density [29]. LAPD measurements may be particularly useful for agents that are administered directly to the lungs, where the nose may not serve as an appropriate proxy. For example, the effect of viral infections, intra-tracheally delivered gene therapy vectors, or inhalational molecules that target the lower airways for delivery all may be better served by evaluation of LAPD, as compared to NPD [25, 26]. Parallel efforts analyzing the electrophysiology of ferret tracheal explants confirmed PD findings, and provide a means to substantiate *in vivo* effects using PD with more definitive Isc results.

To enable *in vitro* analysis of electrophysiological mechanisms that cannot be replicated *in-vivo*, or is more efficient to test *ex vivo*, we report a method to grow differentiated ferret airway epithelia at ALI, and then measure ion transport activity using modified Ussing chambers. Evaluation of primary bronchial epithelial cells in parallel to tissue samples and *in vivo* studies has proven a powerful means to establish novel mechanisms and *in vivo* relevance in CF pigs [16–18], and the methods shown here should be suitable for similar studies of this sort. While a variety of techniques can be used to encourage terminal differentiation of airway epithelial cells in a manner that allows the measurement of vectoral ion transport [30, 31], we focused these initial studies using culture methods frequently reported in the literature for humans and other species [32]; further refinements unique to ferrets may be beneficial to explore in the future. This may be particularly important for evaluating differences in ENaC expression (e.g. alpha, beta, gamma, delta channel subunits) or function, which can vary by species with respect to altered CFTR function [33]. Whether differences in ATP12A expression and function, which has been shown to be crucial for airway acidification in humans and pigs but absent in mice, is another relevant question [17].

Establishing techniques for the study of ferrets may be particularly beneficial for aspects of airway disease that are not represented in mice. For example, even for a monogenetic disease such as CF, mouse models developed by knocking out the causative gene CFTR across a variety of genetic backgrounds failed to recapitulate spontaneous lung disease [34]. Similarly, mouse models of COPD, including those generated by exposing to cigarette smoke and/or exogenous protease administration or introducing genetic deficiencies in anti-proteases, develop emphysematous small airway enlargement, but exhibit minimal alterations in the other pathologic features, especially in conducting airways [35]. Additionally, this study along with previous studies of ion transport in ferrets will help develop more suitable avenues to investigate to specific pathological processes which are more demonstrable in the ferret model of airway disease [36, 37]. Many of the airway disorders have the potential to benefit from a high-order mammal which more faithfully recapitulates human disease, and may be impacted by changes in ion transport. Our hope is that establishing the assays presented here will help enable research of this sort, advancing our understanding of these common and important conditions.

## Supporting information

S1 Fig CT scan of a ferret head. Sagittal reconstructed CT scan image of a ferret head showing the location for the placement of the nasal cannula for measuring nasal potential difference in ferrets.
(TIFF)

S1 Table Solutions for potential difference measurements. Solutions used in the measurement of potential difference listed in the sequential order in which they are perfused.
(DOCX)

## References

[1] Gohy ST, Hupin C, Pilette C, Ladjemi MZ. Chronic inflammatory airway diseases: the central role of the epithelium revisited. Clin Exp Allergy. 2016;46(4):529–42. doi: 10.1111/cea.12712 27021118 10.1111/cea.12712

[2] Mannino DM, Gagnon RC, Petty TL, Lydick E. Obstructive lung disease and low lung function in adults in the United States: data from the National Health and Nutrition Examination Survey, 1988–1994. Arch Intern Med. 2000;160(11):1683–9. 10847262 10.1001/archinte.160.11.1683

[3] Flisikowska T, Kind A, Schnieke A. Genetically modified pigs to model human diseases. J Appl Genet. 2014;55(1):53–64. doi: 10.1007/s13353-013-0182-9 24234401 10.1007/s13353-013-0182-9

[4] Keiser NW, Engelhardt JF. New animal models of cystic fibrosis: what are they teaching us? Curr Opin Pulm Med. 2011;17(6):478–83. 21857224 10.1097/MCP.0b013e32834b14c9 PMC3596000

[5] Fisher JT, Zhang Y, Engelhardt JF. Comparative biology of cystic fibrosis animal models. Methods Mol Biol. 2011;742:311–34. doi: 10.1007/978-1-61779-120-8_19 21547741 10.1007/978-1-61779-120-8_19 PMC3617920

[6] Vlahos R, Bozinovski S. Preclinical murine models of Chronic Obstructive Pulmonary Disease. Eur J Pharmacol. 2015;759:265–71. doi: 10.1016/j.ejphar.2015.03.029 25818750 10.1016/j.ejphar.2015.03.029

[7] Fricker M, Deane A, Hansbro PM. Animal models of chronic obstructive pulmonary disease. Expert Opin Drug Discov. 2014;9(6):629–45. doi: 10.1517/17460441.2014.909805 24754714 10.1517/17460441.2014.909805

[8] Grubb BR, Paradiso AM, Boucher RC. Anomalies in ion transport in CF mouse tracheal epithelium. Am J Physiol. 1994;267(1 Pt 1):C293–300. 8048488 10.1152/ajpcell.1994.267.1.C293

[9] Grubb BR, Boucher RC. Pathophysiology of gene-targeted mouse models for cystic fibrosis. Physiol Rev. 1999;79(1 Suppl):S193–214. 9922382 10.1152/physrev.1999.79.1.S193

[10] Sun X, Sui H, Fisher JT, Yan Z, Liu X, Cho HJ, et al Disease phenotype of a ferret CFTR-knockout model of cystic fibrosis. J Clin Invest. 2010;120(9):3149–60. doi: 10.1172/JCI43052 20739752 10.1172/JCI43052 PMC2929732

[11] Sun X, Olivier AK, Liang B, Yi Y, Sui H, Evans TI, et al Lung phenotype of juvenile and adult cystic fibrosis transmembrane conductance regulator-knockout ferrets. Am J Respir Cell Mol Biol. 2014;50(3):502–12. doi: 10.1165/rcmb.2013-0261OC 24074402 10.1165/rcmb.2013-0261OC PMC4068938

[12] Plourde JR, Pyles JA, Layton RC, Vaughan SE, Tipper JL, Harrod KS. Neurovirulence of H5N1 infection in ferrets is mediated by multifocal replication in distinct permissive neuronal cell regions. PLoS One. 2012;7(10):e46605 doi: 10.1371/journal.pone.0046605 23056366 10.1371/journal.pone.0046605 PMC3466300

[13] Bouvier NM. Animal models for influenza virus transmission studies: a historical perspective. Curr Opin Virol. 2015;13:101–8. doi: 10.1016/j.coviro.2015.06.002 26126082 10.1016/j.coviro.2015.06.002 PMC4550509

[14] Radigan KA, Misharin AV, Chi M, Budinger GS. Modeling human influenza infection in the laboratory. Infect Drug Resist. 2015;8:311–20. doi: 10.2147/IDR.S58551 26357484 10.2147/IDR.S58551 PMC4560508

[15] Raju SV, Kim H, Byzek SA, Tang LP, Trombley JE, Jackson P, et al A ferret model of COPD-related chronic bronchitis. JCI Insight. 2016;1(15):e87536 doi: 10.1172/jci.insight.87536 27699245 10.1172/jci.insight.87536 PMC5033751

[16] Tang XX, Ostedgaard LS, Hoegger MJ, Moninger TO, Karp PH, McMenimen JD, et al Acidic pH increases airway surface liquid viscosity in cystic fibrosis. J Clin Invest. 2016;126(3):879–91. doi: 10.1172/JCI83922 26808501 10.1172/JCI83922 PMC4767348

[17] Shah VS, Meyerholz DK, Tang XX, Reznikov L, Abou Alaiwa M, Ernst SE, et al Airway acidification initiates host defense abnormalities in cystic fibrosis mice. Science. 2016;351(6272):503–7. doi: 10.1126/science.aad5589 26823428 10.1126/science.aad5589 PMC4852973

[18] Hoegger MJ, Fischer AJ, McMenimen JD, Ostedgaard LS, Tucker AJ, Awadalla MA, et al Cystic fibrosis. Impaired mucus detachment disrupts mucociliary transport in a piglet model of cystic fibrosis. Science. 2014;345(6198):818–22. doi: 10.1126/science.1255825 25124441 10.1126/science.1255825 PMC4346163

[19] Solomon GM, Konstan MW, Wilschanski M, Billings J, Sermet-Gaudelus I, Accurso F, et al An international randomized multicenter comparison of nasal potential difference techniques. Chest. 2010;138(4):919–28. doi: 10.1378/chest.10-0179 20472865 10.1378/chest.10-0179 PMC2951758

[20] Van Goor F, Hadida S, Grootenhuis PD, Burton B, Cao D, Neuberger T, et al Rescue of CF airway epithelial cell function in vitro by a CFTR potentiator, VX-770. Proc Natl Acad Sci U S A. 2009;106(44):18825–30. doi: 10.1073/pnas.0904709106 19846789 10.1073/pnas.0904709106 PMC2773991

[21] Pyle LC, Ehrhardt A, Mitchell LH, Fan L, Ren A, Naren AP, et al Regulatory domain phosphorylation to distinguish the mechanistic basis underlying acute CFTR modulators. Am J Physiol Lung Cell Mol Physiol. 2011;301(4):L587–97. doi: 10.1152/ajplung.00465.2010 21724857 10.1152/ajplung.00465.2010 PMC3191754

[22] Rowe SM, Clancy JP, Wilschanski M. Nasal potential difference measurements to assess CFTR ion channel activity. Methods Mol Biol. 2011;741:69–86. doi: 10.1007/978-1-61779-117-8_6 21594779 10.1007/978-1-61779-117-8_6 PMC3760477

[23] Saussereau EL, Roussel D, Diallo S, Debarbieux L, Edelman A, Sermet-Gaudelus I. Characterization of nasal potential difference in cftr knockout and F508del-CFTR mice. PLoS One. 2013;8(3):e57317 doi: 10.1371/journal.pone.0057317 23505426 10.1371/journal.pone.0057317 PMC3591431

[24] Dransfield MT, Wilhelm AM, Flanagan B, Courville C, Tidwell SL, Raju SV, et al Acquired Cystic Fibrosis Transmembrane Conductance Regulator Dysfunction in the Lower Airways in COPD. Chest. 2013;144(2):498–506. doi: 10.1378/chest.13-0274 23538783 10.1378/chest.13-0274 PMC3734887

[25] Alton E, Armstrong DK, Ashby D, Bayfield KJ, Bilton D, Bloomfield EV, et al A randomised, double-blind, placebo-controlled trial of repeated nebulisation of non-viral cystic fibrosis transmembrane conductance regulator (CFTR) gene therapy in patients with cystic fibrosis. Efficacy and Mechanism Evaluation Southampton (UK) 2016. 27441329

[26] Rowe SM, Reeves G, Hathorne H, Solomon GM, Abbi S, Renard D, et al Reduced sodium transport with nasal administration of the prostasin inhibitor camostat in subjects with cystic fibrosis. Chest. 2013;144(1):200–7. doi: 10.1378/chest.12-2431 23412700 10.1378/chest.12-2431 PMC3707174

[27] Fulcher ML, Randell SH. Human nasal and tracheo-bronchial respiratory epithelial cell culture. Methods Mol Biol. 2013;945:109–21. doi: 10.1007/978-1-62703-125-7_8 23097104 10.1007/978-1-62703-125-7_8

[28] Rowe SM, Liu B, Hill A, Hathorne H, Cohen M, Beamer JR, et al Optimizing nasal potential difference analysis for CFTR modulator development: assessment of ivacaftor in CF subjects with the G551D-CFTR mutation. PLoS One. 2013;8(7):e66955 doi: 10.1371/journal.pone.0066955 23922647 10.1371/journal.pone.0066955 PMC3724869

[29] Davies JC, Davies M, McShane D, Smith S, Chadwick S, Jaffe A, et al Potential difference measurements in the lower airway of children with and without cystic fibrosis. Am J Respir Crit Care Med. 2005;171(9):1015–9. doi: 10.1164/rccm.200408-1116OC 15640364 10.1164/rccm.200408-1116OC

[30] Mou H, Vinarsky V, Tata PR, Brazauskas K, Choi SH, Crooke AK, et al Dual SMAD Signaling Inhibition Enables Long-Term Expansion of Diverse Epithelial Basal Cells. Cell Stem Cell. 2016;19(2):217–31. doi: 10.1016/j.stem.2016.05.012 27320041 10.1016/j.stem.2016.05.012 PMC4975684

[31] Suprynowicz FA, Upadhyay G, Krawczyk E, Kramer SC, Hebert JD, Liu X, et al Conditionally reprogrammed cells represent a stem-like state of adult epithelial cells. Proc Natl Acad Sci U S A. 2012;109(49):20035–40. doi: 10.1073/pnas.1213241109 23169653 10.1073/pnas.1213241109 PMC3523865

[32] Neuberger T, Burton B, Clark H, Van Goor F. Use of primary cultures of human bronchial epithelial cells isolated from cystic fibrosis patients for the pre-clinical testing of CFTR modulators. Methods in molecular biology. 2011;741:39–54. doi: 10.1007/978-1-61779-117-8_4 21594777 10.1007/978-1-61779-117-8_4

[33] Tuggle KL, Birket SE, Cui X, Hong J, Warren J, Reid L, et al Characterization of Defects in Ion Transport and Tissue Development in Cystic Fibrosis Transmembrane Conductance Regulator (CFTR)-Knockout Rats. PLoS One. 2014;9(3):e91253 doi: 10.1371/journal.pone.0091253 24608905 10.1371/journal.pone.0091253 PMC3946746

[34] Kent G, Oliver M, Foskett JK, Frndova H, Durie P, Forstner J, et al Phenotypic abnormalities in long-term surviving cystic fibrosis mice. Pediatr Res. 1996;40(2):233–41. doi: 10.1203/00006450-199608000-00008 8827771 10.1203/00006450-199608000-00008

[35] Nikula KJ, Green FH. Animal models of chronic bronchitis and their relevance to studies of particle-induced disease. Inhalation toxicology. 2000;12 Suppl 4:123–53. 12881890 10.1080/089583700750019549

[36] Fisher JT, Tyler SR, Zhang Y, Lee BJ, Liu X, Sun X, et al Bioelectric characterization of epithelia from neonatal CFTR knockout ferrets. Am J Respir Cell Mol Biol. 2013;49(5):837–44. doi: 10.1165/rcmb.2012-0433OC 23782101 10.1165/rcmb.2012-0433OC PMC3931095

[37] Corrales RJ, Coleman DL, Jacoby DB, Leikauf GD, Hahn HL, Nadel JA, et al Ion transport across cat and ferret tracheal epithelia. J Appl Physiol (1985). 1986;61(3):1065–70. 3759745 10.1152/jappl.1986.61.3.1065

