# Supplementary figures and images for: Use of ferrets for electrophysiologic monitoring of ion transport
Source: PLoS One. 2017 Oct 27;12(10):e0186984. doi: 10.1371/journal.pone.0186984 (PMC5659650; doi:10.1371/journal.pone.0186984)

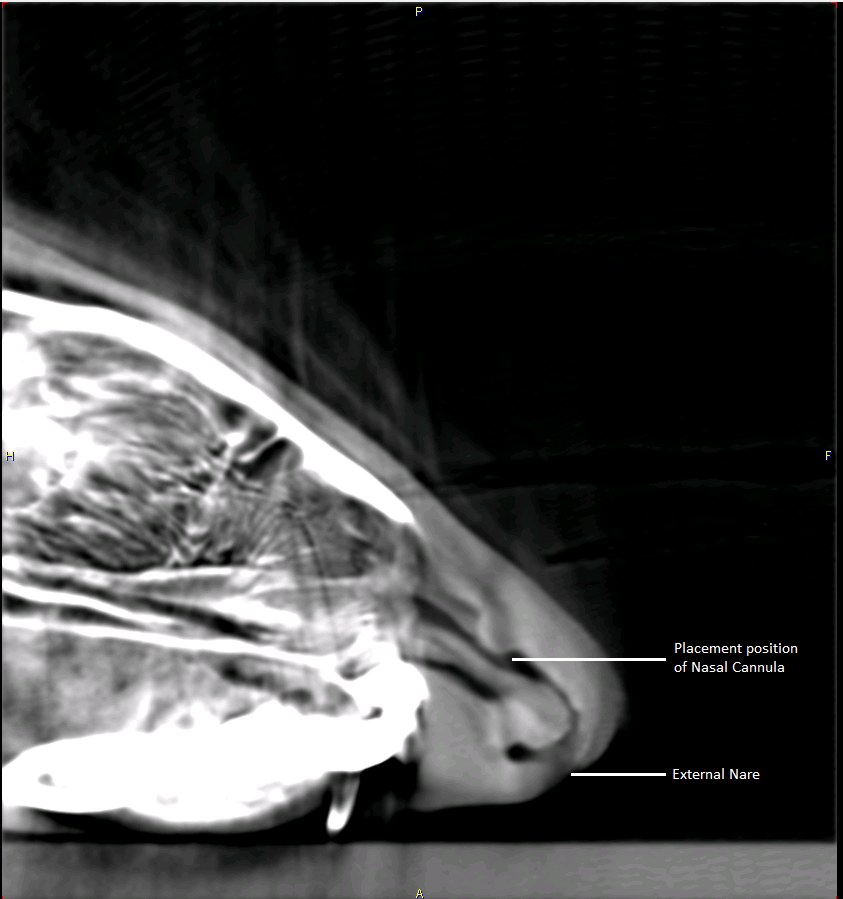

Supplement: S1 Fig — Sagittal reconstructed CT scan image of a ferret head showing the location for the placement of the nasal cannula for measuring nasal potential difference in ferrets. (TIFF) [file pone.0186984.s001.tiff]
