# Supplementary material for: Use of ferrets for electrophysiologic monitoring of ion transport
Source: PLoS One. 2017 Oct 27;12(10):e0186984. doi: 10.1371/journal.pone.0186984 (PMC5659650; doi:10.1371/journal.pone.0186984)
Supplement: S1 Table — Solutions used in the measurement of potential difference listed in the sequential order in which they are perfused. (DOCX) [file pone.0186984.s002.docx]

**S1 Table. Solutions for potential difference measurements**

| **Solution Sequence** | **Perfusate** | **Composition** |
| --- | --- | --- |
| 1 | Ringer’s Irrigation USP | Na^+^ 147 mM,  K^+^ 4 mM,  Ca^++^ 4.5 mM,  Cl^-^ 156 mM |
| 2 | Amiloride | Amiloride (100 µM) plus Solution 1 above |
| 3 | Chloride-free Ringers | K_2_HPO_4_ (2.4 mM)  KH_2_PO_4_ (0.4 mM)  Na Gluconate (115 mM) NaHCO_3_ (25 mM)  Ca Gluconate (1.24 mM) |
| 4 | Forskolin | Forskolin (20 µM) plus Solution 3 above |
| 5 | GlyH101 | GlyH101 (10 µM), plus Solution 4 above |
